# The Elias University Hospital Approach: A Visual Guide to Ultrasound-Guided Botulinum Toxin Injection in Spasticity: Part I—Distal Upper Limb Muscles

**DOI:** 10.3390/toxins17030107

**Published:** 2025-02-26

**Authors:** Marius Nicolae Popescu, Claudiu Căpeț, Cristina Beiu, Mihai Berteanu

**Affiliations:** 1 Department of Physical and Rehabilitation Medicine—Elias Emergency University Hospital, Carol Davila University of Medicine and Pharmacy, 020021 Bucharest, Romania; marius.popescu@umfcd.ro; 2 Clinic of Physical and Rehabilitation Medicine—Elias Emergency University Hospital, 011461 Bucharest, Romania; claudiu.capet@gmail.com; 3 Department of Oncologic Dermatology—Elias Emergency University Hospital, Carol Davila University of Medicine and Pharmacy, 020021 Bucharest, Romania

**Keywords:** post-stroke spasticity, botulinum toxin-A injections, ultrasound-guided therapy, distal upper limb muscles, musculoskeletal ultrasound

## Abstract

Post-stroke spasticity significantly impairs upper limb function and quality of life. Ultrasound-guided botulinum toxin-A (BoNT-A) injections have become a cornerstone of management, enhancing precision and safety. This paper offers a comprehensive guide for clinicians on ultrasound-guided BoNT-A injections for distal upper limb muscles. Each muscle is detailed in terms of its role in spasticity management, ultrasound identification with key anatomical landmarks, clinical relevance, and injection strategies. Motor points, traditionally identified through anatomical studies or electromyography (EMG), are precisely localized using a musculoskeletal ultrasound by targeting the point of maximum muscle thickness, often corresponding to the motor point. The authors present their clinical method, developed at Elias University Hospital (EUH), to refine BoNT-A injection practices. This approach enhances efficacy, reduces dosage requirements, and improves patient outcomes. The paper also explores unique ultrasound characteristics of spastic muscles, such as their relationship with peripheral nerves, adjacent vascular and muscular structures, and intra- and intermuscular fascia, to guide clinicians in targeting functional muscle tissue. This guide is illustrated with representative ultrasound images and clinical diagrams and provides practical insights into anatomical relationships and injection techniques. Part I focuses on distal upper limb muscles, with Part II addressing proximal upper limb muscles.

## 1. Introduction

Post-stroke spasticity is a prevalent and challenging condition that profoundly impacts upper limb function and quality of life [1]. Botulinum toxin-A (BoNT-A) injections have become a cornerstone in its management, providing relief from hypertonia and enhancing motor function. However, the precision required for effective toxin delivery underscores the critical importance of imaging techniques, particularly musculoskeletal ultrasound [2,3].

Ultrasound-guided BoNT-A injections offer significant advantages over traditional techniques, such as manual needle placement or anatomical guidance. Ultrasound provides real-time visualization of muscles, nerves, and vascular structures, ensuring precise targeting of the motor points and avoiding adjacent structures. This reduces the risk of complications, such as inadvertent neurovascular injury, and enhances the effectiveness of the treatment by ensuring accurate toxin delivery into the desired muscle regions [2]. Also, ultrasound allows for dynamic assessments, enabling clinicians to confirm muscle function and contraction during injection, which is particularly valuable for complex anatomical regions like the distal upper limb [4]. Additionally, ultrasound guidance can optimize the cost–benefit ratio of BoNT-A therapy by reducing the required dose. By ensuring accurate localization of the motor point and optimal toxin placement, clinicians can achieve therapeutic outcomes with smaller doses, thereby improving the efficiency and sustainability of the treatment [5].

This paper serves as a practical guide for clinicians performing ultrasound-guided BoNT-A injections in patients with post-stroke spasticity. It focuses specifically on the distal upper limb muscles, offering a structured approach that includes their role in spasticity management, ultrasound identification with key anatomical landmarks, clinical relevance, and injection strategies.

The authors present their clinical method, developed and refined at Elias University Hospital, for targeting spastic muscles with precision. Starting from motor points identified in the literature through anatomical studies or electromyography (EMG) as the sites of densest innervation and most effective stimulation, the authors utilize musculoskeletal ultrasound to precisely locate these motor points by identifying the point of maximum muscle thickness, which often corresponds to the motor point. This approach enhances the accuracy and efficacy of BoNT-A injections. Additionally, the paper discusses the unique ultrasound characteristics of spastic muscles, including the distinctive features of each individual muscle, their relationship with peripheral nerves, adjacent muscular and vascular structures, and specific intra- and intermuscular fascia. These insights guide clinicians in effectively targeting functional muscle tissue, further optimizing treatment outcomes. By sharing this experience, the authors aimed to standardize practices and advance the precision of ultrasound-guided BoNT-A therapy.

This paper represents Part I of a comprehensive guide, focusing on the distal upper limb muscles. Part II will address the proximal upper limb muscles, providing clinicians with a complete framework for performing ultrasound-guided BoNT-A injections to manage post-stroke spasticity effectively.

## 2. Distal Upper Limb Muscles Implicated in Post-Stroke Spasticity

### 2.1. Pronator Teres (PT)

#### 2.1.1. Overview

The pronator teres (PT) is frequently targeted in the management of upper limb spasticity due to its role in resisting passive forearm supination. Spasticity in the PT contributes significantly to the characteristic patterns of hypertonia observed in the upper limb, impeding supination and limiting functional mobility [6].

#### 2.1.2. Ultrasound Identification

Ultrasound identification includes the following: in our clinical practice, the PT is identified using ultrasound by placing the transducer transversely on the volar aspect of the forearm, approximately 2–3 cm distal to the elbow crease on the radial side [7]. Medial to the radial cortex, two muscle masses are visible:

- The PT, which appears oval-shaped on the lateral side;
- The flexor carpi radialis (FCR), which appears triangular-shaped on the medial side [8].

To aid identification, the PT can be visualized as resembling a “ball”, while the FCR appears as a “shark”—a relationship often described as a “shark eating a ball” [7].

#### 2.1.3. Key Ultrasound Landmarks (Figure 1)

The key ultrasound landmarks include the following:

- Muscle position: The PT is the first muscle mass from radial to ulnar on the volar aspect of the forearm [9].
- Intramuscular tendon: In the lateral portion of the muscle, it is positioned longitudinally along the muscle at this level.
- Internal fascia: The fascia separates the humeral (PTHH) and ulnar heads (PTUH) of the PT, while the external fascia distinctly demarcates the PT from adjacent muscle masses, facilitating precise BoNT-A injection.
- Median nerve: The nerve is located deep to the PT, approximately 2 cm distal to the elbow crease. Dynamic evaluation cranially towards the arm reveals the nerve transitioning to the lateral aspect of the muscle alongside the brachial artery [10].
- Dynamic evaluation (Video S1): Scanning proximally toward the medial epicondyle shows an increase in the PT muscle belly size and a concurrent decrease in the size of the FCR. At this level, the intramuscular fascia specific to the PT becomes evident, separating the two muscle heads—the humeral head and the ulnar head—allowing for individual targeting [11].

**Figure 1:**
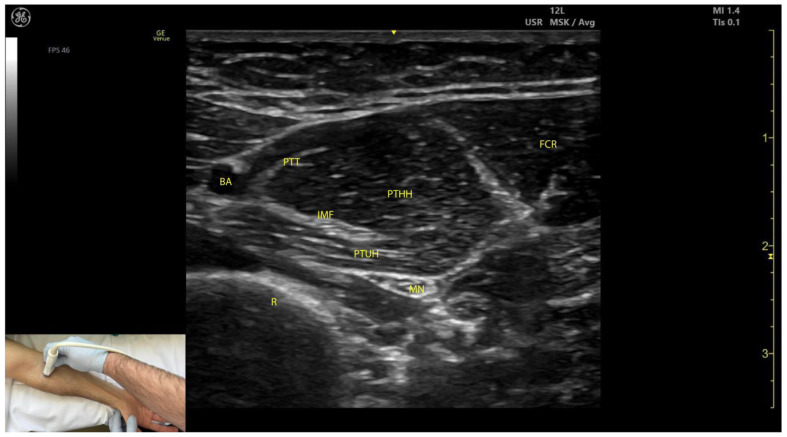
Ultrasound anatomy of the pronator teres (PT) with key landmarks: BA—brachial artery; PT—pronator teres; PTT—pronator teres tendon; R—radius; MN—median nerve; IMF—intramuscular fascia; PTUH—pronator teres ulnar head; PTHH—pronator teres humeral head; FCR—flexor carpi radialis.

#### 2.1.4. Clinical Implications and Injection Strategy

Upper limb spasticity commonly presents with triple flexion patterns: elbow flexion, wrist flexion, finger flexion, and forearm pronation [12]. The therapeutic goal is to restore supination and extension of the involved joints. Functionally, the brachialis is injected as it is the only pure elbow flexor, while the biceps brachii (which also acts as a supinator) and the brachioradialis (which aids in transitioning the forearm from pronation to supination) are preserved to maintain supination capacity [12].

In this context, the PT requires precise BoNT-A targeting:

- The humeral head of the PT should receive a higher dose of BoNT-A because it contributes to elbow flexion [13];
- The ulnar head has a predominant role in pronation of the wrist and does not typically require high-dose injections [14,15,16].

The clinical implications of motor point localization and injection strategy include the following:

- The motor point is located 20% distal to the intercondylar line along the muscle’s length, as described by Sung-Yoon Won et al. [17];
- In our clinical practice, to accurately target this motor point, we employ ultrasound to identify the point of maximum muscle thickness, which aligns closely with the motor point due to its innervation density. BoNT-A injections are then administered with the transducer placed transversely on the volar forearm, approximately 2–3 cm distal to the elbow crease in the radial region.

### 2.2. Flexor Carpi Radialis (FCR)

#### 2.2.1. Overview

The flexor carpi radialis (FCR) is frequently targeted in the management of upper limb spasticity, particularly in cases where resistance is observed during passive wrist extension and adduction maneuvers [18].

#### 2.2.2. Ultrasound Identification

Ultrasound identification includes the following: in our clinical practice, the FCR is visualized using ultrasound by placing the transducer transversely on the volar aspect of the forearm, approximately 4–5 cm distal to the elbow crease on the radial side. Medial to the radial cortex, two muscle masses can be identified: (i) the oval-shaped PT laterally and (ii) the triangular-shaped FCR medially [19].

#### 2.2.3. Key Ultrasound Landmarks (Figure 2)

Key ultrasound landmarks include the following:

- Muscle position: The FCR is the second muscle mass from radial to ulnar on the volar aspect of the forearm [20].
- Intramuscular tendon: The bipennate structure of the FCR is evident on ultrasound due to the presence of longitudinal intramuscular tendon [21].
- Median nerve: The median nerve is initially located deep to the PT, approximately 2 cm distal to the elbow crease. With distal scanning, the nerve transitions into the deeper plane of the FCR and maintains this relationship until the mid-forearm. Beyond this level, the median nerve passes deep to the flexor digitorum superficialis (FDS) [10].
- External fascia: The external fascia distinctly demarcates the FCR from adjacent muscle masses, facilitating precise BoNT-A injection.
- Dynamic evaluation: Muscle contraction is clearly visible during wrist flexion and abduction maneuvers. Scanning distally reveals an increase in the FCR muscle belly size while the PT decreases in size [11].

**Figure 2:**
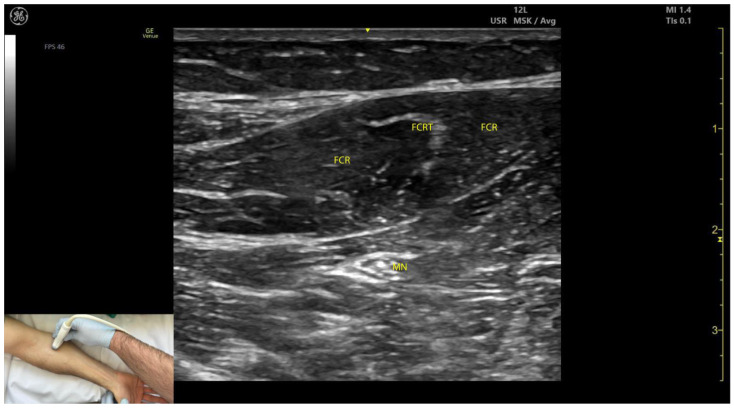
Ultrasound anatomy of the flexor carpi radialis (FCR) with key landmarks: FCR—flexor carpi radialis; FCRT—flexor carpi radialis tendon; MN—median nerve.

#### 2.2.4. Clinical Implications and Injection Strategy

Targeting the FCR with BoNT-A is critical in addressing spasticity that limits wrist extension and functional hand positioning.

The clinical implications of motor point localization and injection strategy include the following:

- A 2014 study investigating the localization of motor points and intramuscular nerve branches of wrist flexors relative to bony landmarks found that the highest concentration of motor points in the FCR is situated approximately 27% along the line connecting the most prominent point of the medial epicondyle to the base of the second metacarpal bone, equivalent to roughly one-fourth of the muscle’s length [22].
- In our clinical practice, ultrasound is used to precisely target the motor point by identifying the point of maximum muscle thickness. The transducer is placed transversely on the volar aspect of the forearm, approximately 4–5 cm distal to the elbow crease in the radial region, ensuring accurate and effective delivery.

### 2.3. Flexor Carpi Ulnaris (FCU)

#### 2.3.1. Overview

The flexor carpi ulnaris (FCU) is frequently targeted in the management of upper limb spasticity, particularly in cases where resistance is encountered during passive wrist extension and abduction maneuvers [23].

#### 2.3.2. Ultrasound Identification

In our clinical practice, the FCU is visualized by placing the transducer transversely on the volar aspect of the forearm, approximately 3–4 cm distal to the elbow crease on the ulnar side. It appears as an oval-shaped muscle superficial to the flexor digitorum profundus (FDP) and the ulnar cortical bone. The FCU is recognized as the strongest wrist flexor [23].

#### 2.3.3. Key Ultrasound Landmarks (Figure 3)

The key ultrasound landmarks include the following:

- Muscle position: The FCU is the most medial muscle mass in the flexor compartment of the wrist, located on the volar aspect of the forearm [24].
- Internal fascia: The intramuscular fascia, often referred to as “cloud fascia” due to its cloud-like appearance, separates the humeral and ulnar heads of the FCU. This fascia can be visualized from the muscle’s origin at the medial epicondyle, extending distally until the FCU transitions into its tendon [23].
- Ulnar nerve (Video S2): The ulnar nerve is located deep to the FCU approximately 2–3 cm distal to the elbow crease. When scanning cranially toward the medial epicondyle, the ulnar nerve can be seen passing between the humeral and ulnar heads of the FCU as it enters the volar compartment of the forearm [25,26]. Scanning distally reveals a gradual decrease in FCU muscle size and an increase in FDP muscle size. At the mid-forearm level, the ulnar nerve joins the ulnar artery, and both structures descend together into the distal forearm toward Guyon’s canal [26].
- External fascia: The external fascia distinctly demarcates the FCU from adjacent muscle masses, facilitating precise BoNT-A injection.
- Dynamic evaluation: Contraction of the FCU is observed during wrist flexion and adduction maneuvers.

**Figure 3:**
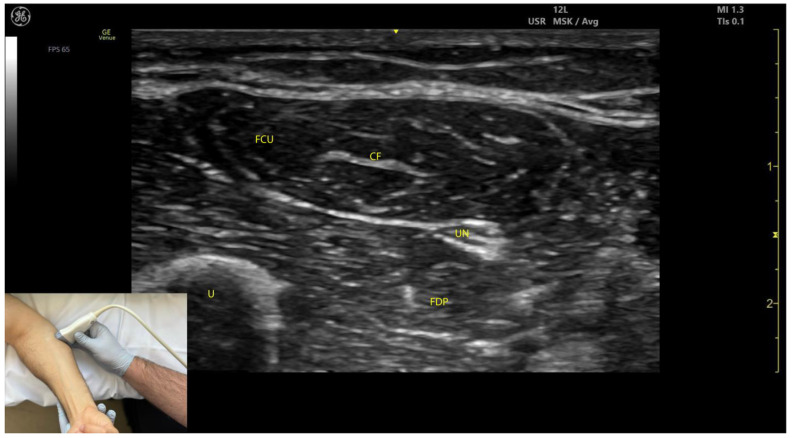
Ultrasound anatomy of the flexor carpi ulnaris (FCU) with key landmarks: FCU—flexor carpi ulnaris; UN—ulnar nerve; FDP—flexor digitorum profundus; U—ulna; CF—cloud fascia.

#### 2.3.4. Clinical Implications and Injection Strategy

The clinical implications of motor point localization and injection strategy include the following:

- Electromyographic studies indicate that the region with the highest concentration of motor plates in the FCU is located approximately 32% along the line connecting the most prominent point of the medial epicondyle to the pisiform bone, corresponding to about one-third of the muscle’s length [22].
- In our clinical practice, ultrasound is used to precisely target the motor point by identifying the point of maximum muscle thickness. The transducer is positioned transversely on the volar aspect of the forearm, approximately 4 cm distal to the elbow crease in the ulnar portion, ensuring accurate and effective delivery of BoNT-A.

### 2.4. Flexor Digitorum Superficialis (FDS)

#### 2.4.1. Overview

The flexor digitorum superficialis (FDS) is frequently targeted in the management of upper limb spasticity, particularly in patterns involving resistance to passive extension of the wrist and the metacarpophalangeal and proximal interphalangeal joints of fingers II–V [27].

#### 2.4.2. Ultrasound Identification

In our clinical practice, the FDS is visualized using ultrasound by placing the transducer transversely on the volar aspect of the forearm at the midline (approximately half the forearm length). It is located in the intermediate layer of the anterior compartment, superficial to the flexor digitorum profundus (FDP) and deep to the FCR and FCU [28].

#### 2.4.3. Key Ultrasound Landmarks (Figure 4 and Figure 5)

The key ultrasound landmarks include the following:

- Muscle position: At the mid-forearm, the FDS represents the second muscle mass from the cortical surfaces of the radius and ulna, moving from deep to superficial. Superficial to the FDS are the lateral FCR and medial FCU [29]. In the absence of US guidance, these adjacent muscles may be mistaken for the FDS, increasing the risk of injection errors.
- Median nerve: The median nerve is adhered to the deep surface of the FDS. At this level, the FDS and FDP are separated by the intermuscular fascia, which houses the median nerve, ulnar nerve, and ulnar artery [26].
- External fascia: FDS2 and FDS3 have a pronounced external fascia that distinctly demarcates them from adjacent muscle masses during BoNT-A injections, whereas FDS4 and FDS5 lack this feature.
- Dynamic evaluation (Video S3): The contraction of FDS muscle bellies corresponding to fingers II–V is observed during flexion of the metacarpophalangeal and proximal interphalangeal joints. A paradoxical arrangement of FDS2 and FDS3 bellies can be noted anatomically, as they appear inversely positioned medially and laterally relative to the median nerve [30]. Dynamic scanning proximal to the medial epicondyle reveals that the maximal thickness of FDS4 is located approximately 2–3 cm distal to the elbow crease, on the volar aspect of the forearm in the ulnar portion. At this level, the FDS4 is situated medial to the FCR and lateral to the FCU, following the line connecting the medial epicondyle to the pisiform bone [31]. A potential source of error in targeting FDS4 at this level is the palmaris longus (PL) muscle, which, if present, is located between the FCR and FDS4 muscle bellies. The PL is variably present in 74–97.5% of the population and is most commonly bilateral [32,33].

**Figure 4:**
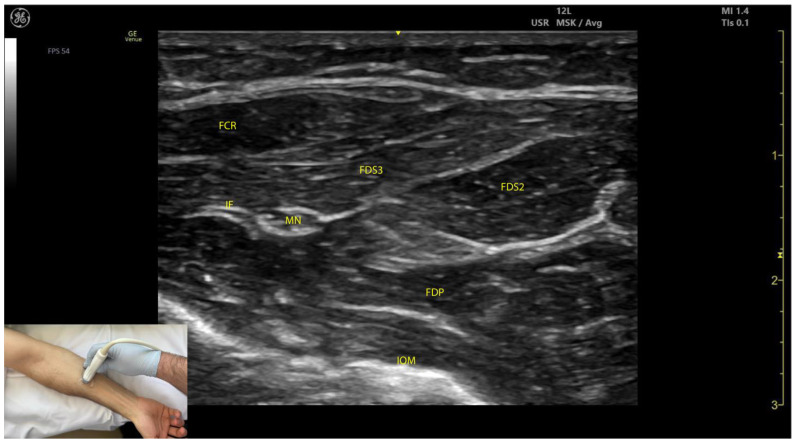
Ultrasound anatomy of the flexor digitorum superficialis (FDS) with key landmarks: FCR—flexor carpi radialis; FDS3—flexor digitorum superficialis digit 3; FDS2—flexor digitorum superficialis digit 2; IF—intermuscular fascia; FDP—flexor digitorum profundus; IOM—interosseous membrane; MN—median nerve.

**Figure 5:**
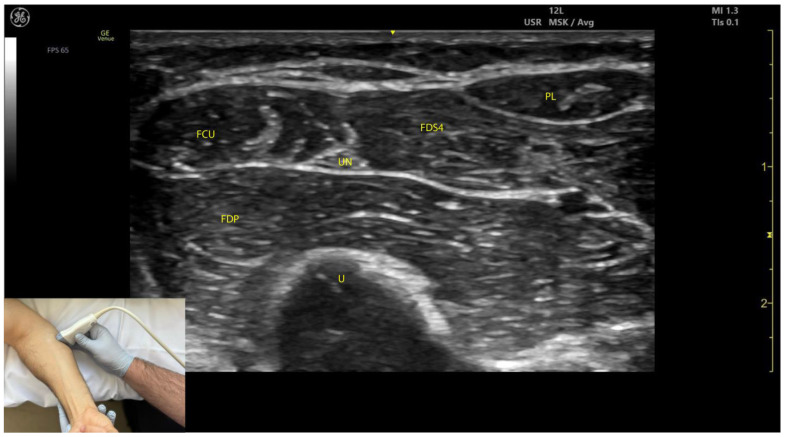
Ultrasound anatomy of the flexor digitorum superficialis (FDS4) with key landmarks: FDS4—flexor digitorum superficialis digit 4; PL—palmaris longus; FCU—flexor carpi ulnaris; U—ulna; UN—ulnar nerve; FDP—flexor digitorum profundus.

#### 2.4.4. Clinical Implications and Injection Strategy

Ultrasound guidance is crucial for the accurate localization and targeting of the FDS, particularly given its anatomical relationships with adjacent muscles (FCR and FCU) and neurovascular structures (median nerve and ulnar artery).

The clinical implications of motor point localization and injection strategy include the following:

- The motor point of the FDS is located 30% along the interepicondylar-interstyloidar line in the humeroulnar portion and 50% along the interepicondylar-interstyloidar line in the radial portion [34].
- In our clinical practice, we target the FDS for BoNT-A injections at the point of maximum muscle thickness as determined by ultrasound:
  1. For FDS2, FDS3, and FDS5, this site is typically located at the volar aspect of the mid-forearm;
  2. For FDS4, this site is typically located 2–3 cm distal to the elbow crease in the ulnar portion of the forearm.

Both in-plane and out-of-plane ultrasound-guided techniques can be utilized. In-plane means the needle is inserted along the ultrasound beam, so its full length is visible during the injection. Out-of-plane means the needle crosses the ultrasound beam, appearing as a dot, allowing access in tight spaces but with less visualization of the entire needle [35]. We prefer the in-plane technique which allows simultaneous injection of FDS2, FDS3, and FDS5 at their points of maximum thickness, reducing the number of local injections required and improving patient adherence to treatment.

### 2.5. Flexor Digitorum Profundus (FDP)

#### 2.5.1. Overview

The flexor digitorum profundus (FDP) is frequently targeted in the management of upper limb spasticity, particularly in patterns involving resistance to passive extension of the distal interphalangeal joints of fingers II–V [36].

#### 2.5.2. Ultrasound Identification

In our clinical practice, the FDP is visualized using ultrasound by placing the transducer transversely on the volar aspect of the forearm, at approximately half the forearm length. It is located in the deep layer of the anterior compartment, superficial to the cortical surfaces of the radius and ulna and deep to the FDS [37]. The FDP is responsible for flexing the distal interphalangeal (DIP) joints of fingers II–V (FDP 2–5). Each muscle belly corresponds to a specific finger (index, middle, ring, and little fingers). Importantly, the thumb’s DIP joint is flexed by a separate muscle, the flexor pollicis longus (FPL), which is anatomically and functionally distinct from the FDP [36]. Therefore, targeting the FDP inherently refers to FDP2–5.

#### 2.5.3. Key Ultrasound Landmarks (Figure 4 and Figure 5)

The key ultrasound landmarks include the following:

- Muscle position: At the mid-forearm, the FDP is the first muscle mass located directly superficial to the cortical surfaces of the radius and ulna [29].
- Intermuscular fascia: The FDP is separated from the FDS by intermuscular fascia that houses the median nerve, ulnar nerve, and ulnar artery [31].
- Median nerve: The median nerve adheres to the deep surface of the FDS and is positioned superficially relative to the FDP at this level [31].
- Internal fascia: FDP 2–5 is a single continuous muscle with four distinct tendons, corresponding to digits 2–5. Unlike some other muscles, the FDP does not have a pronounced fascia that demarcates separate muscle masses for each individual finger, making it a continuous muscular structure without clear fascial boundaries for each digit during BoNT-A injection.
- Dynamic evaluation (Video S4): Contraction of the FDP is observed during isolated flexion of the distal interphalangeal joints of fingers II–V, with proximal interphalangeal joints held in a blocked position [29].

#### 2.5.4. Clinical Implications and Injection Strategy

The FDP is crucial for finger flexion, particularly at the DIP joints. Precise localization during BoNT-A injections is essential to effectively reduce spasticity while avoiding adjacent structures, such as the median and ulnar nerves [36].

The clinical implications of motor point localization and injection strategy include the following:

- The motor point of the FDP is located 40% along the interepicondylar-interstyloidar line along the muscle in the medial portion and 60% along the interepicondylar-interstyloidar line along the muscle in the lateral portion [34].
- In our practice, BoNT-A is administered into the point of maximum muscle thickness of the FDP as determined by ultrasound. For FDP 2–5, the optimal site is typically located on the volar aspect of the mid-forearm. The in-plane technique is often preferred, as it facilitates a single injection for FDP 2–5 at their points of maximum muscle thickness, reducing the number of injection sites and improving patient adherence. The out-of-plane technique can also be employed for targeted injections into individual muscle bellies when necessary.

### 2.6. Flexor Pollicis Longus (FPL)

#### 2.6.1. Overview

The flexor pollicis longus (FPL) is frequently targeted in the management of upper limb spasticity, particularly in patterns involving resistance during passive extension of the thumb at the metacarpophalangeal (MCP) and interphalangeal (IP) joints [38].

#### 2.6.2. Ultrasound Identification

In our clinical practice, the FPL is visualized using ultrasound by placing the transducer transversely on the volar aspect of the forearm, in the radial portion of the distal third. Medial to the radial cortex, the FPL appears as a distinctive muscle, often described as having a “shark fin” shape, located in the deep layer of the anterior compartment of the forearm [7].

#### 2.6.3. Key Ultrasound Landmarks (Figure 6)

The key ultrasound landmarks include the following:

- Muscle position: The FPL is the first muscle mass medial to the radial cortex in the distal third of the forearm [38].
- Radial artery: The radial artery lies superficial to the FPL.
- Median nerve: The median nerve is positioned medial to the muscle [38].
- External fascia: The FPL muscle lacks a pronounced fascia to clearly separate it from adjacent muscle masses, such as the FDS, FDP, and FCR, which can make precise localization more challenging during BoNT-A injections.
- Dynamic evaluation (Video S5): Scanning distally toward the wrist, the FPL muscle tapers and transitions into its tendon near the radial styloid [39]. Muscle contraction is observed during flexion of the thumb at the MCP and IP joints.

**Figure 6:**
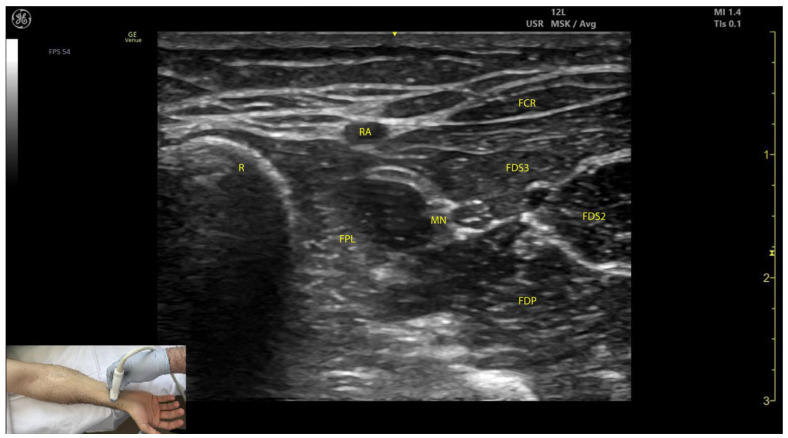
Ultrasound anatomy of the flexor pollicis longus (FPL) with key landmarks: FPL—flexor pollicis longus; R—radius; MN—median nerve; RA—radial artery; FDS3—flexor digitorum superficialis digit 3; FDS2—flexor digitorum superficialis digit 2; FDP—flexor digitorum profundus.

#### 2.6.4. Clinical Implications and Injection Strategy

Reducing spasticity in the FPL through BoNT-A injections is a primary therapeutic goal, particularly in cases of the “thumb-in-hand” deformity, which can lead to palmar ulcerations. This treatment improves fine motor function and overall quality of life for patients [40]. The close proximity of the radial artery and the median nerve, as well as the radial cortex, presents a challenge. The distance between these structures is often less than 1 cm, requiring precise ultrasound guidance to avoid complications [38].

The clinical implications of motor point localization and injection strategy include the following:

- The motor points of the FLP are located at 50% and 65% along the intercondylar-interstyloidar line [34].
- In our clinical practice, BoNT-A is injected at the point of maximum muscle thickness of the FPL, typically located in the radial portion of the distal third of the forearm. The out-of-plane technique is typically employed due to the limited space between anatomical structures. The injection is performed through one of two ultrasound windows: between the radial artery and the radial cortex or between the radial artery and the median nerve. Both approaches require real-time ultrasound guidance to ensure accurate placement of the toxin within the muscle, prevent injury to the median nerve and radial artery, and minimize the risk of local hematoma formation.

### 2.7. Pronator Quadratus (PQ)

#### 2.7.1. Overview

The pronator quadratus (PQ) is frequently targeted in the management of upper limb spasticity, particularly for resistance during passive supination of the forearm [12].

#### 2.7.2. Ultrasound Identification

In our clinical practice, the PQ is visualized using ultrasound by placing the transducer transversely on the volar aspect of the forearm, approximately 3–4 cm proximal to the distal radioulnar joint. It is located superficial to the bony cortices of the radius and ulna and spans medially and laterally relative to these bones [41].

#### 2.7.3. Key Ultrasound Landmarks (Figure 7)

The key ultrasound landmarks include the following:

- Muscle position: The PQ is the deepest muscle on the anterior forearm [31];
- Quadrilateral shape: It has a distinct, rectangular shape with parallel muscle fibers [41];
- Unique fiber orientation: The PQ is the only forearm muscle with fibers oriented perpendicularly to the limb, unlike all other forearm muscles, which run longitudinally;
- Two heads: The PQ has superficial and deep heads, separated by an intramuscular fascia that connects the convex surfaces of the radius and ulna [41];
- Deep to the PQ: There is the interosseous membrane and the extensor compartment of the distal forearm [42];
- Superficial to the PQ: There is the FDP, FDS, and FPL tendons, the median nerve, and, if present, the palmaris longus tendon [42];
- External fascia: The PQ muscle is characterized by a well-defined fascia that clearly separates it from adjacent muscle masses, facilitating precise localization during BoNT-A injections.
- Dynamic evaluation: When scanning distally toward the radioulnar joint, the PQ muscle enlarges, while the FDP, FPL, and FDS progressively decrease in size and transition into tendons [43].

**Figure 7:**
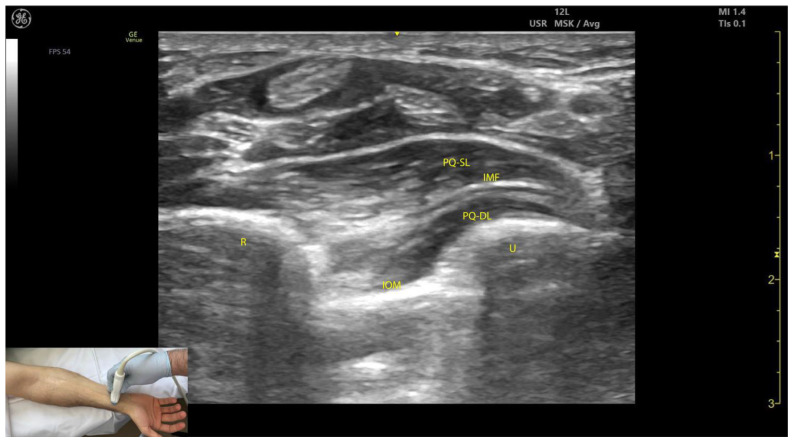
Ultrasound anatomy of the pronator quadratus (PQ) with key landmarks: PQ-SL—pronator quadratus superficial layer; PQ-DL—pronator quadratus deep layer; IOM—interosseous membrane; IMF—intramuscular fascia.

#### 2.7.4. Clinical Implications and Injection Strategy

Targeting the PQ is crucial for reducing spasticity that restricts forearm supination and for improving overall limb function. By stabilizing the wrist joint, the PQ plays a key role in maintaining proper wrist alignment during forearm pronation and supination. Its function includes displacing the ulna proximally and preventing the ulnar head from impinging on the carpal bones, which is essential for coordinated wrist and forearm movements [44].

In clinical practice, the PQ is frequently injected with BoNT-A to reduce spasticity, particularly when agonists such as the pronator teres resist supination. Relaxing the PQ not only alleviates resistance to supination but also enhances the function of antagonist muscles, including the biceps brachii and supinator, leading to improved forearm mobility and overall limb functionality [45].

The clinical implications of motor point localization and injection strategy include the following:

- According to Choung et al., the optimal injection site for the PQ, containing the highest concentration of motor points, is approximately 3 cm proximal to the ulnar styloid [46].
- In our clinical practice, the BoNT-A injections are performed at the point of maximum muscle thickness, as determined by ultrasound. The PQ can be injected either through the volar aspect of the forearm, avoiding the median nerve, or through the dorsal aspect via the extensor compartment and interosseous membrane. Both approaches require ultrasound guidance to ensure accurate delivery while avoiding neurovascular structures and minimizing the risk of complications.

### 2.8. Abductor Pollicis Brevis (APB)

#### 2.8.1. Overview

The abductor pollicis brevis (APB) is targeted in the management of hand spasticity particularly for resistance during passive adduction of the thumb at the MCP joint [47].

#### 2.8.2. Ultrasound Identification

In our clinical practice, the APB is visualized using ultrasound by placing the transducer transversely on the palmar aspect of the hand at the base of the thenar eminence. It appears superficial to the bony cortex of the first metacarpal and is fusiform in shape. The opponens pollicis is located deep to the APB [48].

#### 2.8.3. Key Ultrasound Landmarks (Figure 8)

The key ultrasound landmarks include the following:

- Muscle position: The APB is the first muscle mass from lateral to medial within the thenar eminence, also being the most superficial muscle mass of the thenar eminence [49].
- External fascia: The APB muscle lacks a pronounced fascia to clearly separate it from adjacent muscle masses, such as the opponens pollicis and flexor pollicis brevis, which can complicate precise localization during BoNT-A injections.
- Dynamic evaluation (Video S6): Scanning proximally toward the radial styloid shows an increase in the size of the APB, while the size of the opponens pollicis decreases. Muscle contraction of the APB is visible during thumb abduction at the MCP joint [50].

**Figure 8:**
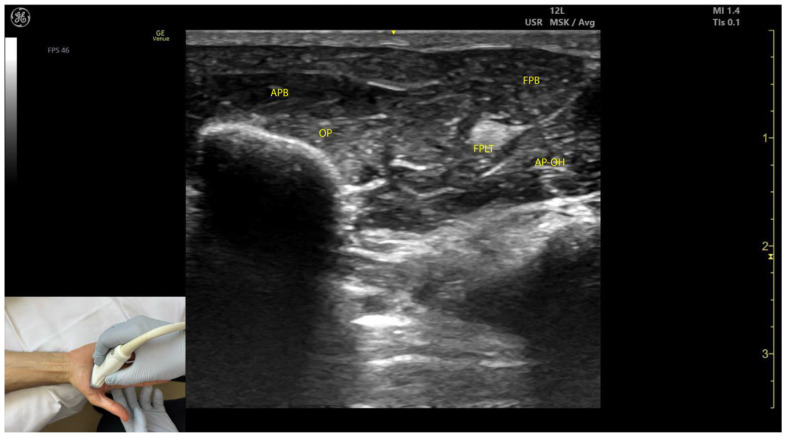
Ultrasound anatomy of the abductor pollicis brevis (APB) with key landmarks: APB—abductor pollicis brevis; OP—opponens pollicis; FPB—flexor pollicis brevis; AP-OH adductor pollicis oblique head; FPLT—flexor pollicis longus tendon.

#### 2.8.4. Clinical Implications and Injection Strategy

Addressing spasticity in the APB can significantly improve thumb mobility and overall hand function in patients with hand spasticity. However, the limited availability of detailed data on APB spasticity in the literature underscores the need for further studies to better understand its role and optimize treatment strategies [50].

The clinical implications of motor point localization and injection strategy include the following:

- The region with the highest density of intramuscular nerve arborizations of the APB is located 40% along the line connecting the hook of hamate to the head of the first metacarpal [51].
- In our clinical practice, the APB is targeted for BoNT-A injections at the point of maximum muscle thickness, determined via ultrasound, typically located at the base of the thenar eminence.

### 2.9. Opponens Pollicis (OP)

#### 2.9.1. Overview

The opponens pollicis (OP) is frequently targeted in hand spasticity patterns, particularly for resistance during passive adduction and lateral rotation of the carpometacarpal (CMC) joint of the thumb, as well as extension of the thumb at the MCP joint [52].

#### 2.9.2. Ultrasound Identification

In our clinical practice, the OP is visualized using ultrasound by placing the transducer transversely on the palmar aspect of the hand, at the midpoint of the thenar eminence. It appears deep to the APB and has a triangular shape. The OP lies superficial to the cortex of the first metacarpal [48].

#### 2.9.3. Key Ultrasound Landmarks (Figure 8)

The key ultrasound landmarks include the following:

- Muscle position: The OP is the second structure from superficial to deep within the thenar eminence [52].
- Muscle size: It is the largest muscle within the thenar eminence [53].
- Lateral position: The OP is located lateral to the flexor pollicis brevis (FPB) [53].
- External fascia: The OP muscle lacks a pronounced fascia to distinctly separate it from adjacent muscle masses, such as the abductor pollicis brevis and flexor pollicis brevis, which may make precise localization during BoNT-A injections more challenging.
- Dynamic evaluation: Scanning distally toward the MCP joint shows an increase in the size of the OP and a corresponding decrease in the size of the APB. At this level, the tendon of the FPL can be seen deep to the OP [54]. Muscle contraction of the OP is observed during adduction and medial rotation of the thumb at the CMC joint, flexion at the MCP joint, or during thumb opposition when the tip of the thumb contacts the fifth finger [55].

#### 2.9.4. Clinical Implications and Injection Strategy

Targeting the OP with BoNT-A improves thumb mobility and alignment in spasticity patterns, enhancing hand function and dexterity.

The clinical implications of motor point localization and injection strategy include the following:

- The region with the highest density of intramuscular nerve arborizations of the opponens is located 60% along the line connecting the hook of hamate to the head of the first metacarpal [51].
- In our clinical practice, BoNT-A injections into the OP are performed at the point of maximum muscle thickness, as determined by ultrasound. The optimal injection site is typically located approximately 1 cm proximal to the MCP joint on the palmar aspect of the hand. Ultrasound guidance ensures accurate delivery while avoiding adjacent structures, such as the flexor pollicis longus tendon.

### 2.10. Flexor Pollicis Brevis (FPB)

#### 2.10.1. Overview

The flexor pollicis brevis (FPB) is frequently targeted in the management of “thumb-in-hand” spasticity patterns. This condition involves resistance during passive extension of the thumb at the CMC and MCP joints [56,57].

#### 2.10.2. Ultrasound Identification

In our clinical practice, the FPB is visualized using ultrasound by placing the transducer transversely on the palmar aspect of the hand at the thenar eminence. It is located medial to the APB and superficial to the cortical surface of the first metacarpal [48].

#### 2.10.3. Key Ultrasound Landmarks (Figure 9)

The key ultrasound landmarks include the following:

- Muscle position: The FPB is the most medial muscle mass within the thenar eminence. It is situated medial to the APB and opponens pollicis and lateral to the adductor pollicis [56].
- Internal fascia: The FPB contains intramuscular fascia that separates its two heads—the superficial and deep heads—which can be targeted individually during injections [53];
- FPL tendon: Scanning toward the MCP joint reveals the FPL tendon located lateral to the superficial head of the FPB and deep to its deep head.
- External fascia: The FPB muscle lacks a pronounced fascia to clearly separate it from adjacent muscle masses, such as the abductor pollicis brevis and opponens pollicis, which may complicate precise localization during BoNT-A injections.
- Dynamic evaluation: Contraction of the FPB is observed during thumb flexion at the CMC and MCP joints [48].

**Figure 9:**
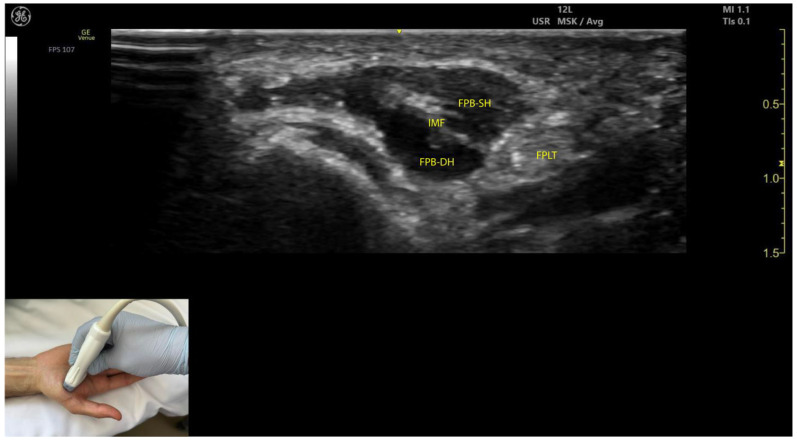
Ultrasound anatomy of the flexor pollicis brevis (FPB) with key landmarks: FPB-SH—flexor pollicis brevis superficial head; FPB-DH—flexor pollicis brevis deep head; IMF—intramuscular fascia; FPLT—flexor pollicis longus tendon.

#### 2.10.4. Clinical Implications and Injection Strategy

The FPB is one of the primary muscles involved in the “thumb-in-hand” spasticity pattern seen in pediatric patients with cerebral palsy. This condition interferes with local hygiene, increases the risk of skin lesions, and significantly impairs hand functions such as grasp, pinch, and release [57].

The clinical implications of motor point localization and injection strategy include the following:

- The region with the highest density of intramuscular nerve arborizations of the FPB is located 50–70% along the line connecting the hook of hamate to the head of the first metacarpal [51].
- In our clinical practice, the FPB is targeted for BoNT-A injections at the point of maximum muscle thickness, as determined by ultrasound. The optimal injection site is typically located at the midpoint of the thenar eminence. Ultrasound guidance ensures precise toxin delivery and allows for individual targeting of the superficial and deep heads, minimizing the risk of complications.

### 2.11. Adductor Pollicis (AP)

#### 2.11.1. Overview

The adductor pollicis (AP) is frequently targeted in hand spasticity patterns involving resistance to passive thumb abduction at the CMC and MCP joints. This resistance significantly impacts grasp and digital pinch functions [58].

#### 2.11.2. Ultrasound Identification

In our clinical practice, the AP is visualized using ultrasound by placing the transducer transversely on the palmar aspect of the hand at the midpoint of the second and third metacarpals. It appears as a triangular muscle located superficial to the cortical surfaces of the second and third metacarpals [48].

#### 2.11.3. Key Ultrasound Landmarks (Figure 10 and Figure 11)

The key ultrasound landmarks include the following:

- Muscle size: The AP is larger than the muscles of the thenar eminence [58].
- Unique anatomical compartment: The AP is the only muscle in the adductor compartment of the hand [59].
- Two heads: The AP consists of a transverse head and an oblique head: (i) The transverse head is identified with the transducer placed transversely on the palmar aspect at the midpoint of the second and third metacarpals. Scanning proximally (approximately 1 cm) reveals the muscle’s maximal thickness; (ii) the oblique head is visualized by angling the transducer approximately 45 degrees laterally toward the thumb. This head is located lateral to the FPL tendon and the FPB, with the first dorsal interosseous muscle in its depth [59,60];
- External fascia: The AP muscle is characterized by a pronounced fascia that clearly separates it from adjacent muscle masses, facilitating precise localization during BoNT-A injections.
- Dynamic evaluation: Contraction of the AP is observed during thumb adduction at the CMC and MCP joints or during pinch movements, such as bringing the thumb tip into contact with the index finger tip.

**Figure 10:**
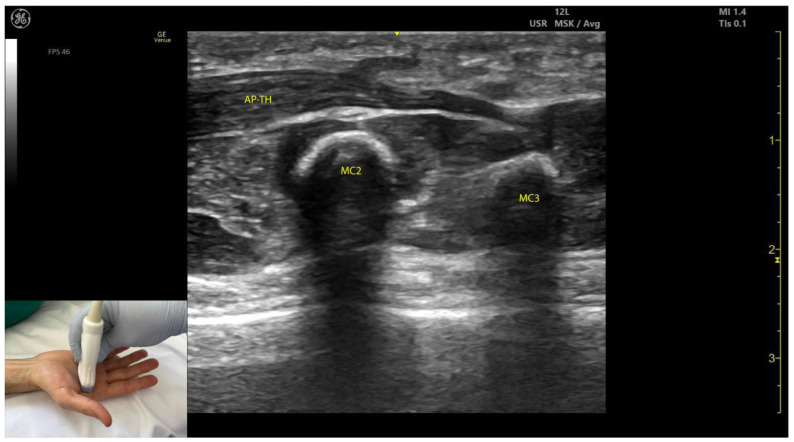
Ultrasound anatomy of the adductor pollicis transverse head (AP-TH) with key landmarks: AP-TH adductor pollicis transverse head; MC2—second metacarpal; MC3—third metacarpal.

**Figure 11:**
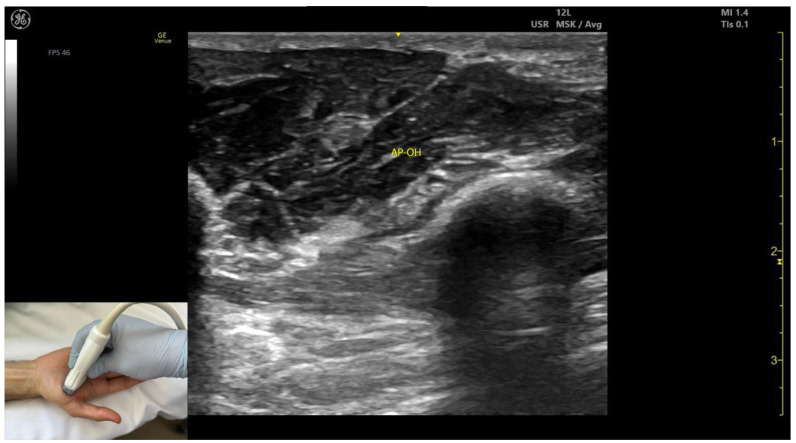
Ultrasound anatomy of the adductor pollicis oblique head (AP-OH) with key landmarks: AP-OH—adductor pollicis oblique head.

#### 2.11.4. Clinical Implications and Injection Strategy

The clinical implications and injection strategy include the following:

- The AP is commonly injected with BoNT-A due to its critical role in thumb adduction, a movement it performs alongside the FPB and OP [61]. According to a 2023 study by Yi et al., the highest concentration of motor endplates is located on 1/5–3/5 of the muscle from the midline of the third metacarpal bone to the base of the first proximal phalanx [61].
- In our clinical practice, BoNT-A is administered into the point of maximum muscle thickness, as determined by ultrasound. The injection is typically performed approximately 1 cm proximal to the midpoint of the second and third metacarpals, with the transducer angled 45 degrees toward the thumb to target both the transverse and oblique heads.

### 2.12. Lumbricals (L)

#### 2.12.1. Overview

The lumbricals are frequently targeted in hand spasticity patterns, particularly for resistance during passive extension of the fingers at the MCP joints and flexion of the fingers at the IP joints of digits II–V [62,63].

#### 2.12.2. Ultrasound Identification

In our clinical practice, the lumbrical muscles are visualized using ultrasound by placing the transducer transversely on the palmar aspect of the hand, below the thenar eminence, at the midpoint of the metacarpals corresponding to digits II–V. The palmar interossei muscles I–III are located between the cortical surfaces of metacarpals II–V. Superficial to the cortical surfaces of metacarpals II–III, the transverse head of the AP is visualized. Moving from radial to ulnar, the following structures are identified: L1, FDS2, and FDP2; L2, FDS3, and FDP3; L3, FDS4, and FDP4; and L4, FDS5, and FDP5 [48]. The lumbricals are also recognized by their distinct oval shape [62].

#### 2.12.3. Key Ultrasound Landmarks [63,64,65,66] **(**Figure 12)

The key ultrasound landmarks include the following:

- Muscle morphology: Lumbricals L1 and L2 are unipennate, while L3 and L4 are bipennate. Intramuscular fascia corresponding to L1 and L2 can be visualized.
- Innervation and vascular supply: Superficial to L1 and L2 are branches of the median nerve, while superficial to L3 and L4 are branches of the ulnar nerve, each accompanied by arterial branches from the dorsal carpal arch.
- External fascia: The lumbrical muscles lack a pronounced fascia to distinctly separate them from the FDS tendons (adjacent structures), which may pose challenges for precise localization during BoNT-A injections.
- Dynamic evaluation: Scanning distally toward the MCP joints reveals an increase in the size of L1–L4 and a corresponding decrease in the size of the AP. Placing the transducer at the midpoint of the metacarpals II–V and performing flexion and extension maneuvers of the fingers highlights the contraction of L1–L4 and the associated action on the tendons of FDS and FDP. The lumbricals appear as hypoechoic structures with hyperechoic speckles, described as a “starry sky” pattern, while the FDS and FDP tendons are hyperechoic with parallel fibrillar lines.

**Figure 12:**
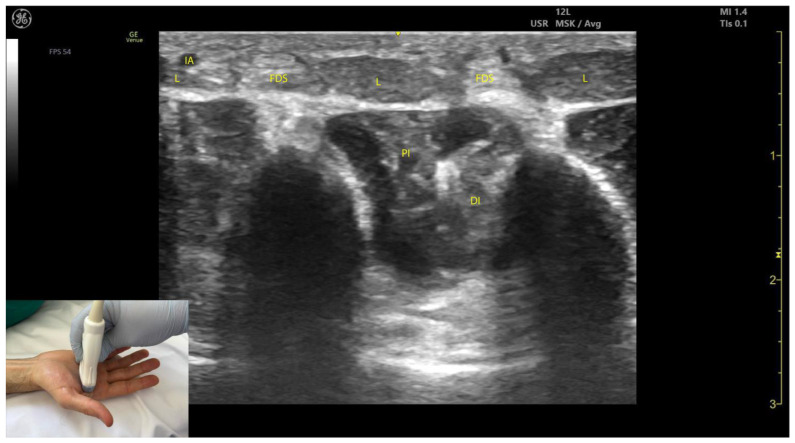
Ultrasound anatomy of the lumbricals (L) with key landmarks: L—lumbricals; PI—palmar interossei; DI—dorsal interossei; IA—interdigital artery; FDS—flexor digitorum superficialis.

#### 2.12.4. Clinical Implications and Injection Strategy

Targeting the lumbricals is crucial in reducing spasticity that restricts finger movements, improving both hand function and patient quality of life [67]. In our clinical practice, BoNT-A injections into the lumbricals are administered at the point of maximum muscle thickness, as determined by ultrasound. The optimal injection site is typically located approximately 1 cm proximal to the MCP joints of digits II–V.

### 2.13. Interossei Muscles

#### 2.13.1. Overview

The interossei muscles are frequently targeted in hand spasticity patterns, often in conjunction with the lumbricals, to address resistance during passive finger movements. These include adduction, flexion, and extension in the case of the dorsal interossei and abduction, flexion, and extension of fingers II, IV, and V in the case of the palmar interossei [68,69]

#### 2.13.2. Ultrasound Identification

In our clinical practice, the interossei muscles are visualized using ultrasound by placing the transducer transversely on the dorsal aspect of the hand, 1–2 cm proximal to the MCP joints of the fingers. Alternatively, the transducer can be placed longitudinally on the dorsal aspect of the hand, aligned with the palpable bony landmarks of the metacarpals I–V. Four dorsal interossei muscles are identified between the cortical surfaces of the metacarpals I–V, while three palmar interossei muscles are located deeper, between the cortical surfaces of metacarpals II–V [48].

#### 2.13.3. Key Ultrasound Landmarks [60,67,70] (Figure 13)

The key ultrasound landmarks include the following:

- Muscle position: The dorsal interossei are the most superficial muscle structures on the dorsal aspect of the hand.
- Muscle morphology: The dorsal interossei are bipennate muscles, whereas the palmar interossei are unipennate. Musculoskeletal ultrasound also allows visualization of the intramuscular fascia within these structures.
- External fascia: The interossei muscles lack a pronounced fascia to clearly demarcate the dorsal and palmar interossei (adjacent muscle masses), which can make precise localization during BoNT-A injections more challenging.
- Muscle size: The first dorsal interosseous muscle is larger compared to the other dorsal interossei. Deep to it lies the AP, which decreases in size as the first dorsal interosseous muscle increases during dynamic scanning cranially.

**Figure 13:**
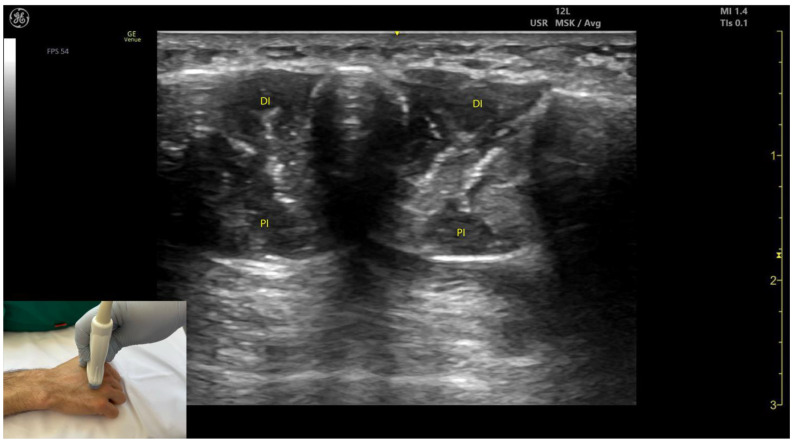
Ultrasound anatomy of the interossei muscles with key landmarks: DI—dorsal interossei; PI—palmar interossei.

#### 2.13.4. Clinical Implications and Injection Strategy

Spasticity of the interossei muscles can lead to complications such as anterior subluxation of the proximal phalanx and flexion of the MCP joint beyond 90 degrees [71]. Addressing spasticity in these muscles with BoNT-A injections can mitigate these complications and restore hand functionality.

A 2023 study demonstrated that ultrasound-guided injections using an in-plane approach with the transducer placed longitudinally on the dorsal hand effectively reduce spasticity and confirm the safety and efficacy of this treatment [72].

In our clinical practice, BoNT-A is administered into the point of maximum muscle thickness of the interossei muscles, as determined by ultrasound. The injections can be performed with the transducer placed transversely on the dorsal aspect of the hand, approximately 1–2 cm proximal to the MCP joints or longitudinally along the dorsal aspect of the hand, between the palpable bony landmarks of the metacarpals I–V.

## 3. Ultrasound Characteristics of Spastic Muscles and Their Clinical Relevance

Spastic muscles exhibit significant structural and echogenic alterations that distinguish them from normal muscles. These changes, which include increased echogenicity, disrupted intramuscular fascia, and alterations in muscle volume, impact both ultrasound imaging and BoNT-A injection strategies.

**Figure 14:**
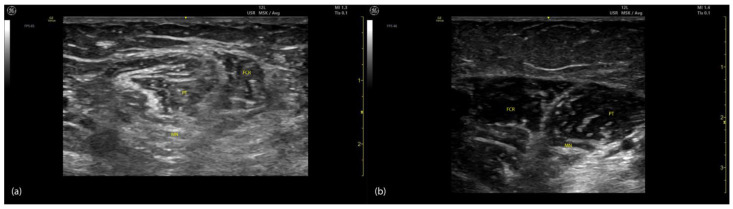
Comparison of ultrasound characteristics in spastic and normal PT and FCR: (**a**) ultrasound image of spastic muscles, including the PT and FCR, showing altered echogenicity and disrupted muscular architecture; (**b**) ultrasound image of the same muscles in a normal state, demonstrating uniform echotexture, clear muscle boundaries, and normal positioning of the MN; PT—pronator teres; FCR—flexor carpi radialis; MN—median nerve.

**Figure 15:**
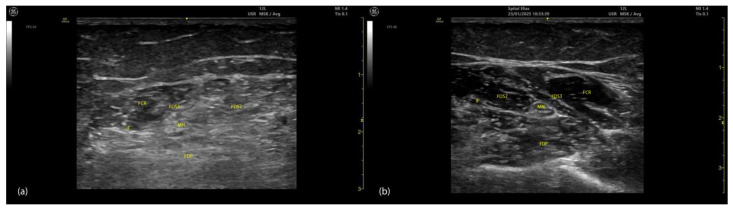
Comparison of ultrasound characteristics in spastic and normal muscles. (**a**) ultrasound image of spastic muscles (e.g., FDS3 and FDS2) showing altered echogenicity, disrupted intermuscular fascia (IF), and structural changes near the median nerve (MN) and adjacent FDP muscle; (**b**) ultrasound image of the same muscles in a normal state, demonstrating uniform echotexture, intact IF, and clear differentiation between muscle layers and the MN; FDS3—flexor digitorum superficialis digit 3; FDS2—flexor digitorum superficialis digit 2; MN—median nerve; IMF—intramuscular fascia; FDP—flexor digitorum profundus; FCR—flexor carpi radialis.

Spasticity induces progressive alterations in muscle architecture, including atrophy, fibrosis, sarcomere loss, and fatty infiltration, which are observable on ultrasound (Figure 14 and Figure 15) [73].

Some of the ultrasound characteristics include the following:

- Atrophy: On ultrasound, spastic muscles often appear smaller, with a reduced cross-sectional area compared to normal muscles. The reduction in muscle volume correlates with decreased contractile strength and functionality, necessitating precise targeting during BoNT-A injections to maximize therapeutic outcomes [69];
- Increased echogenicity: Spastic muscles commonly exhibit increased echogenicity due to intramuscular connective tissue accumulation (fibrosis) and fatty infiltration. This “whiter” appearance on ultrasound indicates pathological changes in the muscle tissue, which can complicate the identification of motor points or regions of maximum muscle activity [74];
- Loss of homogeneous texture: Normal muscles display a striated echotexture due to their uniform fibrous and contractile elements. In contrast, spastic muscles appear heterogeneous, with disrupted striations caused by fibrosis and fatty infiltration. This heterogeneity can make it challenging to differentiate between functional muscle tissue and pathological regions [75];
- Altered dynamic response: Dynamic ultrasound evaluation of spastic muscles often reveals reduced or paradoxical contraction patterns during voluntary or passive movements. This is due to sarcomere loss and shortening, which impair the muscle’s mechanical efficiency and responsiveness to stretch [76,77];
- Increased stiffness: On elastography, spastic muscles show increased stiffness compared to normal muscles. This stiffness reflects both increased connective tissue deposition and muscle hypertonicity, influencing the choice of injection technique and dose distribution [78].

The changes in spastic muscles impact both the identification and treatment strategy for BoNT-A injections. As discussed throughout this paper, identifying the point of maximum muscle activity or tension is crucial for effective BoNT-A delivery. In spastic muscles with heterogeneous architecture, this requires careful evaluation of dynamic and static ultrasound findings. Clinicians must also consider that increased echogenicity from fatty infiltration or fibrosis may indicate non-functional areas, where injections may be less effective, underscoring the importance of targeting viable muscle tissue [76]. Regarding treatment strategy, dose adjustment and injection technique are paramount. Spastic muscles may require higher BoNT-A doses due to increased stiffness and hypertonicity; however, dose distribution should be carefully planned to avoid overtreatment, particularly in muscles with significant fibrosis or fatty infiltration [79]. Additionally, altered muscle architecture necessitates precise needle placement [80]. Techniques such as in-plane or out-of-plane guidance should be selected based on the spatial constraints and the location of motor points within the spastic muscle [35,67]. By recognizing these structural and functional changes, clinicians can refine their injection strategies and optimize treatment outcomes for patients with post-stroke spasticity.

## 4. Conclusions

This paper provides a comprehensive framework for performing ultrasound-guided BoNT-A injections in the distal upper limb muscles for managing post-stroke spasticity. By leveraging a musculoskeletal ultrasound, clinicians can precisely identify the point of maximum muscle thickness, often corresponding to the motor point, enabling accurate toxin delivery while minimizing risks such as neurovascular injury and unintended toxin diffusion. This precision significantly enhances treatment efficacy and patient outcomes.

The authors’ injection model, developed and refined at Elias University Hospital, highlights the importance of integrating anatomical knowledge, ultrasound imaging, and evidence-based practices to optimize spasticity management. The model emphasizes tailoring interventions to each patient’s unique anatomy, reducing the required toxin dose, and improving cost-effectiveness without compromising safety or efficacy.

Furthermore, the paper explores the unique ultrasound characteristics of spastic muscles, such as increased echogenicity, fibrosis, and fatty infiltration, and their implications for injection strategies. Recognizing these structural changes ensures that injections are targeted at functional muscle tissue, avoiding non-viable regions and maximizing therapeutic outcomes.

This work represents Part I of a series focusing on distal upper limb muscles. Part II will address proximal upper limb muscles, providing a complete guide for clinicians managing spasticity across the entire upper limb. Through this comprehensive approach, the authors aim to standardize ultrasound-guided injection techniques, improve clinical outcomes, and advance the field of post-stroke spasticity management.

## Supplementary Materials

The following supporting information can be downloaded at https://doi.org/10.5281/zenodo.14768396, Video S1: dynamic ultrasound evaluation of the pronator teres (PT); Video S2: dynamic ultrasound evaluation of the flexor carpi ulnaris (FCU); Video S3: dynamic ultrasound evaluation of the flexor digitorum superficialis (FDS); Video S4: dynamic ultrasound evaluation of the flexor digitorum profundus (FDP); Video S5: dynamic ultrasound evaluation of the flexor pollicis longus (FPL); Video S6: dynamic ultrasound evaluation of the abductor pollicis brevis (APB).

## Abbreviations

The following abbreviations are used in this manuscript:

BoNT-A	Botulinum Toxin-A
EMG	Electromyography
EUH	Elias University Hospital
PT	Pronator Teres
PTT	Pronator Teres (tendon)
FCR	Flexor Carpi Radialis
PTHH	Pronator Teres Humeral Head
PTUH	Pronator Teres Ulnar Head
FCU	Flexor Carpi Ulnaris
CF	Cloud Fascia
FDS	Flexor Digitorum Superficialis
FDP	Flexor Digitorum Profundus
FPL	Flexor Pollicis Longus
PL	Palmaris Longus
PQ	Pronator Quadratus
PQ-SL	Pronator Quadratus Superficial Layer
PQ-DL	Pronator Quadratus Deep Layer
APB	Abductor Pollicis Brevis
OP	Opponens Pollicis
FPB	Flexor Pollicis Brevis
FPB-SH	Flexor Pollicis Brevis Superficial Head
FPB-DH	Flexor Pollicis Brevis Deep Head
AP	Adductor Pollicis
AP-TH	Adductor Pollicis Transverse Head
AP-OH	Adductor Pollicis Oblique Head
MCP	Metacarpophalangeal (joint)
IP	Interphalangeal (joint)
DIP	Distal Interphalangeal (joint)
CMC	Carpometacarpal (joint)
IMF	Intramuscular Fascia
IF	Intermuscular Fascia
RA	Radial Artery
IA	Interdigital Artery
MN	Median Nerve
UN	Ulnar Nerve
IOM	Interosseous Membrane
PI	Palmar Interossei
DI	Dorsal Interossei
FPLT	Flexor Pollicis Longus (tendon)
R	Radius (bone)
U	Ulna (bone)
MC	Metacarpal (bone)
L	Lumbrical (muscle)
